# Supplementary material for: Hypoxic Preconditioning Promotes Survival of Human Adipose Derived Mesenchymal Stem Cell
Source: F1000Res. 2024 Jul 16;10:843. Originally published 2021 Aug 23. [Version 4] doi: 10.12688/f1000research.55351.4 (PMC11208860; doi:10.12688/f1000research.55351.4)
Supplement: Peer review history

# Reviewer report: Reviewer response for version 4

**DOI:** 10.5256/f1000research.169042.r303635

**Published:** 2024-08-08

**Authors:** Ming Pei

**Affiliations:** 1 Department of Orthopaedics, West Virginia University, Morgantown, WV, 26506-9196, USA

I approve the revision and response letter from the authors. I have not further comments to make. Thanks.

Is the work clearly and accurately presented and does it cite the current literature?

Partly

If applicable, is the statistical analysis and its interpretation appropriate?

I cannot comment. A qualified statistician is required.

Are all the source data underlying the results available to ensure full reproducibility?

Partly

Is the study design appropriate and is the work technically sound?

Partly

Are the conclusions drawn adequately supported by the results?

Partly

Are sufficient details of methods and analysis provided to allow replication by others?

Partly

Reviewer Expertise:

adult stem cell and tissue regeneration

I confirm that I have read this submission and believe that I have an appropriate level of expertise to confirm that it is of an acceptable scientific standard.

# Reviewer report: Reviewer response for version 3

**DOI:** 10.5256/f1000research.167902.r295867

**Published:** 2024-07-11

**Authors:** Ming Pei

**Affiliations:** 1 Department of Orthopaedics, West Virginia University, Morgantown, WV, 26506-9196, USA

The conclusion is not appropriate, which also reflects in the title. I suggest to delete “via expression of prosurvival and proangiogenic biomarkers” from the title. For the results, I did not find (1) the data (Table 3) (Figure 5) to support “VEGF influenced the expression of SCF” (page 8/23), (2) the data (Table 5) (Figure 7) to support “OCT-4 effect on BCL2 expression”, and (3) the data (Table 7) (Figure 9) to support “BCL2 expression inhibited apoptosis” and “HSP27 expression also inhibited apoptosis”.

Is the work clearly and accurately presented and does it cite the current literature?

Partly

If applicable, is the statistical analysis and its interpretation appropriate?

I cannot comment. A qualified statistician is required.

Are all the source data underlying the results available to ensure full reproducibility?

Partly

Is the study design appropriate and is the work technically sound?

Partly

Are the conclusions drawn adequately supported by the results?

Partly

Are sufficient details of methods and analysis provided to allow replication by others?

Partly

Reviewer Expertise:

adult stem cell and tissue regeneration

I confirm that I have read this submission and believe that I have an appropriate level of expertise to confirm that it is of an acceptable scientific standard, however I have significant reservations, as outlined above.

# Response

**Published:** 2024-07-11

**Authors:** Ricardo Adrian Nugraha

**Affiliations:** Airlangga University, Indonesia

The conclusion is not appropriate, which also reflects in the title.

**Authors response:** thank you for your suggestion. We decided to change the conclusion statement according to the results with statement “From this study, it can be concluded that the hypoxic preconditioning  of h-AMSC culture has proven to increase the expression of VEGF, SCF, OCT-4, and BCL2 and HSP27. This study demonstrated and explained the existence of a new mechanism of increased h-AMSC survival in cultures with hypoxic preconditioning (O _2_ 1%) via VEGF, SCF, OCT-4, BCL2, and HSP 27.”

 I suggest to delete “via expression of prosurvival and proangiogenic biomarkers” from the title.

**Authors response:** thank you for your suggestion. We have deleted “via expression of prosurvival and proangiogenic biomarkers” from the title.

 For the results, I did not find (1) the data (Table 3) (Figure 5) to support “VEGF influenced the expression of SCF” (page 8/23), (2) the data (Table 5) (Figure 7) to support “OCT-4 effect on BCL2 expression”, and (3) the data (Table 7) (Figure 9) to support “BCL2 expression inhibited apoptosis” and “HSP27 expression also inhibited apoptosis”.

**Authors response: **thank you for your very critical feedback. After intense discussion with all the authors, we decided to change the statement into “hypoxic preconditioning influenced the expression of SCF (based on Table 3), OCT4 (based on Table 4), BCL2 (based on Table 5), HSP27 expression (based on Table 6) and apoptosis (based on Table 7). We decided to move the Figure 10 (path analysis) in the last subsection of the results section.

# Reviewer report: Reviewer response for version 3

**DOI:** 10.5256/f1000research.167902.r292484

**Published:** 2024-06-26

**Authors:** Suat Cheng Tan

**Affiliations:** 1 Universiti Sains Malaysia, Kelantan, Malaysia

The authors had answered my comments.

Is the work clearly and accurately presented and does it cite the current literature?

Yes

If applicable, is the statistical analysis and its interpretation appropriate?

I cannot comment. A qualified statistician is required.

Are all the source data underlying the results available to ensure full reproducibility?

Partly

Is the study design appropriate and is the work technically sound?

Yes

Are the conclusions drawn adequately supported by the results?

Yes

Are sufficient details of methods and analysis provided to allow replication by others?

Partly

Reviewer Expertise:

Stem cell biology

I confirm that I have read this submission and believe that I have an appropriate level of expertise to confirm that it is of an acceptable scientific standard.

# Reviewer report: Reviewer response for version 2

**DOI:** 10.5256/f1000research.137196.r288323

**Published:** 2024-06-12

**Authors:** Suat Cheng Tan

**Affiliations:** 1 Universiti Sains Malaysia, Kelantan, Malaysia

This manuscript  described the roles of hypoxic preconditioning in promoting survival of human adipose derived mesenchymal stem cell via expression of prosurvival and proangiogenic biomarkers. It is well written, data is sufficient and can be considered for approval if the authors can provide more detail for the methodologies and statistical analysis protocol.

Is the work clearly and accurately presented and does it cite the current literature?

Yes

If applicable, is the statistical analysis and its interpretation appropriate?

I cannot comment. A qualified statistician is required.

Are all the source data underlying the results available to ensure full reproducibility?

Partly

Is the study design appropriate and is the work technically sound?

Yes

Are the conclusions drawn adequately supported by the results?

Yes

Are sufficient details of methods and analysis provided to allow replication by others?

Partly

Reviewer Expertise:

Stem cell biology

I confirm that I have read this submission and believe that I have an appropriate level of expertise to confirm that it is of an acceptable scientific standard, however I have significant reservations, as outlined above.

# Response

**Published:** 2024-06-13

**Authors:** Ricardo Adrian Nugraha

**Affiliations:** Airlangga University, Indonesia

Thank you for your feedback and suggestion. We added several explanation regarding the methodologies, cell cultures subsection and data analysis subsection.

# Reviewer report: Reviewer response for version 2

**DOI:** 10.5256/f1000research.137196.r153685

**Published:** 2024-02-08

**Authors:** Gun-Jae Jeong

**Affiliations:** 1 School of Biological Sciences, Georgia Institute of Technology, Atlanta, GA, USA

I think this manuscript in now acceptable for indexing.

Is the work clearly and accurately presented and does it cite the current literature?

Yes

If applicable, is the statistical analysis and its interpretation appropriate?

Partly

Are all the source data underlying the results available to ensure full reproducibility?

Yes

Is the study design appropriate and is the work technically sound?

Yes

Are the conclusions drawn adequately supported by the results?

Yes

Are sufficient details of methods and analysis provided to allow replication by others?

No

Reviewer Expertise:

Mesenchmal stem cells, tissue engineering

I confirm that I have read this submission and believe that I have an appropriate level of expertise to confirm that it is of an acceptable scientific standard.

# Reviewer report: Reviewer response for version 1

**DOI:** 10.5256/f1000research.58917.r128761

**Published:** 2022-04-27

**Authors:** Gun-Jae Jeong

**Affiliations:** 1 School of Biological Sciences, Georgia Institute of Technology, Atlanta, GA, USA

The article entitled “Hypoxic preconditioning promotes survival of human adipocyte mesenchymal stem cell via expression of prosurvival and proangiogenic biomarkers” by I Gde Rurus Suryawan *et al.* (2021) reported enhanced survival of human adipocyte mesenchymal stem cell with hypoxic preconditioning. This study is well designed, and several experiments are performed well. However, this manuscript needs to be modified in certain minor aspects:

 Minor comments:

1. Please revise the adipocyte mesenchymal stem cell as adipose derived mesenchymal stem cell or other appropriate word. The word adipocyte already contains the meaning of cell (-cyte). Therefore, the meanings are overlapped in a word.
2. Please provide more information of immunocytochemistry methods and antibody information in materials and methods section.
3. Please provide detailed quantification methods for each quantification. What is the measurement of each quantification experiment?
4. Overall figures: please include scale bars for each figure set. It looks like they have different magnifications.
5. Overall figures: please resize each subset figures as same size in one figure.

Is the work clearly and accurately presented and does it cite the current literature?

Yes

If applicable, is the statistical analysis and its interpretation appropriate?

Partly

Are all the source data underlying the results available to ensure full reproducibility?

Yes

Is the study design appropriate and is the work technically sound?

Yes

Are the conclusions drawn adequately supported by the results?

Yes

Are sufficient details of methods and analysis provided to allow replication by others?

No

Reviewer Expertise:

Mesenchmal stem cells, tissue engineering

I confirm that I have read this submission and believe that I have an appropriate level of expertise to confirm that it is of an acceptable scientific standard, however I have significant reservations, as outlined above.

# Response

**Published:** 2022-04-27

**Authors:** Ricardo Adrian Nugraha

**Affiliations:** Airlangga University, Indonesia

Dear Prof. Gun Jae Jeong

 Thank you for your comment and suggestion. We are happy to get valuable comment regarding our work.

 Author Response Letter to Reviewer Comments:

1. Please revise the adipocyte mesenchymal stem cell as adipose derived mesenchymal stem cell or other appropriate word. The word adipocyte already contains the meaning of cell (-cyte). Therefore, the meanings are overlapped in a word. -> We will revised as your suggestion.
2. Please provide more information of immunocytochemistry methods and antibody information in materials and methods section. -> We will provide more information regarding the immunocytochemistry methods and antibody information
3. Please provide detailed quantification methods for each quantification. What is the measurement of each quantification experiment? -> We will provide detailed quantification methods
4. Overall figures: please include scale bars for each figure set. It looks like they have different magnifications. -> Unfortunately, scale bars is difficult to achieve since we got it from the third party
5. Overall figures: please resize each subset figures as same size in one figure. -> We will resize our figure in the same size

# Response

**Published:** 2022-08-07

**Authors:** Ricardo Adrian Nugraha

**Affiliations:** Airlangga University, Indonesia

The article entitled “Hypoxic preconditioning promotes survival of human adipocyte mesenchymal stem cell via expression of prosurvival and proangiogenic biomarkers” by I Gde Rurus Suryawan *et al.* (2021) reported enhanced survival of human adipocyte mesenchymal stem cell with hypoxic preconditioning. This study is well designed, and several experiments are performed well. However, this manuscript needs to be modified in certain minor aspects:

 Minor comments:

1. Please revise the adipocyte mesenchymal stem cell as adipose derived mesenchymal stem cell or other appropriate word. The word adipocyte already contains the meaning of cell (-cyte). Therefore, the meanings are overlapped in a word.

**Author Response: Thank you for your suggestion, author have already replace adipocyte mesenchymal stem cell with adipose derived mesenchymal stem cell**

  

1. Please provide more information of immunocytochemistry methods and antibody information in materials and methods section.

**Author Response: Thank you for your suggestion. We have provided additional information regarding the immunocytochemistry methods and antibody information**

  

1. Please provide detailed quantification methods for each quantification. What is the measurement of each quantification experiment?

**Author Response: We measured mean fluorescence intensity (MFI) in a region of interest (ROI) with ImageJ software. We were simply measuring automatic cell counting based on the total fluorescent intensity across the entire image that lead to the potentially erroneous conclusion that the sample with the smaller area/fewer cells exhibits less staining for the probe of interest than one that is larger in area or has more cells, when the reality is that they actually exhibit similar staining levels per tissue area**

  

1. Overall figures: please include scale bars for each figure set. It looks like they have different magnifications.

**Author Response: Figure 3-10 had similar magnification. Unfortunately, figure 1 and 2 had different magnification. We try to add scale bare but it couldn’t work well.**

  

1. Overall figures: please resize each subset figures as same size in one figure.

**Author Response: Figure 3-10 had been resize as same size in one figure. Unfortunately, we try to resize figure 1 and 2 but it couldn’t be better.**
